# Are Non-cardiac Surgeries Safe for Dialysis Patients? – A Population-Based Retrospective Cohort Study

**DOI:** 10.1371/journal.pone.0058942

**Published:** 2013-03-14

**Authors:** Yih-Giun Cherng, Chien-Chang Liao, Tso-Hsiao Chen, Duan Xiao, Chih-Hsiung Wu, Ta-Liang Chen

**Affiliations:** 1 Department of Anesthesiology, Shuang Ho Hospital, affiliated with Taipei Medical University, New Taipei City, Taiwan; 2 Department of Anesthesiology, School of Medicine, College of Medicine, Taipei Medical University, Taipei, Taiwan; 3 Department of Anesthesiology, Taipei Medical University Hospital, Taipei, Taiwan; 4 Health Policy Research Center, Taipei Medical University Hospital, Taipei, Taiwan; 5 Department of Nephrology, Wan Fang Medical Center, affiliated with Department of Internal Medicine, College of Medicine, Taipei Medical University, Taipei, Taiwan; 6 Department of Coloproctology, the Second People’s Hospital of Shi-Fang City, Shi-Fang City, Sichuan Province, People Republic of China; 7 Department of Surgery, Shuang Ho Hospital, affiliated with Taipei Medical University, New Taipei City, Taiwan; Inserm, France

## Abstract

**Background:**

End-stage renal disease represents a risk complex that complicates surgical results. The surgical outcomes of dialysis patients have been studied in specific fields, but the global features of postoperative adverse outcomes in dialysis patients receiving non-cardiac surgeries have not been examined.

**Methods:**

Taiwan’s National Health Insurance Research Database was used to study 8,937 patients under regular dialysis with 8,937 propensity-score matched-pair controls receiving non-cardiac surgery between 2004 and 2007. We investigated the influence of hemodialysis and peritoneal dialysis, effects of hypertension and diabetes, and impact of additional comorbidities on postoperative adverse outcomes.

**Results:**

Postoperative mortality in dialysis patients was higher than in controls (odds ratio [OR] 3.33, 95% confidence interval [CI] 2.56 to 4.33) when receiving non-cardiac surgeries. Complications such as acute myocardial infarction, pneumonia, bleeding, and septicemia were significantly increased. Postoperative mortality was significantly increased among peritoneal dialysis patients (OR 2.71, 95% CI 1.70 to 4.31) and hemodialysis patients (OR 3.42, 95% CI 2.62 to 4.47) than in controls. Dialysis patients with both hypertension and diabetes had the highest risk of postoperative complications; these risks increased with number of preoperative medical conditions. Patients under dialysis also showed significantly increased length of hospitalization, more ICU stays and higher medical expenditures.

**Conclusion:**

Surgical patients under dialysis encountered significantly higher postoperative complications and mortality than controls when receiving non-cardiac surgeries. Different dialysis techniques, pre-existing hypertension/diabetes, and various comorbidities had complication-specific impacts on surgical adverse outcomes. These findings can help surgical teams provide better risk assessment and postoperative care for dialysis patients.

## Introduction

Renal function impairment is a chronic and progressive process that is usually a complication of hypertension, diabetes mellitus, glomerulonephritis, drug abuse or other etiologies such as heavy metal intoxication [1]–[4]. Advanced renal dysfunction results in end-stage renal disease (ESRD), which may render patients dialysis-dependent. Chronic kidney disease can be considered a pre-ESRD stage whose prevalence during this period in Taiwan was usually underestimated due to lack of public awareness [5], [6]. Health care for patients with chronic kidney disease or ESRD is usually a complicated task due to their diverse etiologies, coexisting diseases, complications and types of dialysis [5], [7]. The issues of patient awareness, socio-economic status, compliance with therapy, vascular accessibility and nutritional and hormonal balance might also further increase the complexity of dialysis patient care [8], [9].

As expected, overall mortality of dialysis-dependent patients is much higher than of non-dialysis populations, especially due to cardiovascular diseases and their complications [1], [10], [11]. Recent improvement in dialysis techniques, such as more frequent dialysis and more effective retained solutes removal, have provided uremic patients with longer survival times and better life quality [12]–[15]. With the prolongation of lifespan, dialysis-dependent patients are more likely to undergo surgical procedures. As for postoperative complications and mortality, most studies were confined to cardiovascular surgeries and procedures [16]–[18]; investigations on other surgeries were few with limited sample sizes [19], [20]. Accordingly, global postoperative outcomes for dialysis patients exposed to non-cardiac surgery on a population-based scale were not well defined.

Taiwan has noted the world’s highest prevalence rate of ESRD during 2001–2007, with a total of 53,242 ESRD subjects undergoing dialysis treatment by 2008 [1] and the estimated prevalence rate of chronic kidney disease for adults aged 20 years and older over the period from 1994 to 2006 was 11.93% [5]. We conducted a retrospective nationwide population-based study among dialysis-dependent surgical patients receiving non-cardiac surgery to illustrate the global features of postoperative complications and mortality, the impact of peritoneal dialysis (PD) or hemodialysis (HD), coexisting with hypertension or diabetes mellitus, and additional medical conditions on postoperative adverse outcomes.

## Methods

### Data Sources and Study Population

This study used reimbursement claims data from Taiwan’s National Health Insurance, a universal insurance program started in March 1995. More than 99% of 22.6 million Taiwan residents are enrolled in this system. Taiwan’s National Health Research Institutes established a National Health Insurance Research Database (NHIRD) to record all beneficiaries’ inpatient and outpatient medical services. Information in the database includes patients’ demographics, primary and secondary disease diagnoses, procedures, prescriptions and medical expenditures. The NHIRD’s validity has been documented [21] and research from this database has been published in our previous work and by many others [22], [23]. For confidentiality, the electronic database was decoded with patient identifications scrambled to protect privacy for further public access. The study was evaluated and approved by the NHIRD research committee.

We examined medical claims and identified 8,937 surgical patients with preoperative regular HD or PD from 2,010,412 persons who underwent major inpatient surgeries between 2004 and 2007. Receiving HD or PD at least three times per week for more than three months before the index surgery was defined as preoperative regular dialysis in this study [24], [25]. We used propensity-score to select matched-controls randomly by age, sex, teaching hospital, coexisting disease, type of surgery and anesthesia from surgical patient populations without a history of preoperative renal dialysis. Major inpatient surgeries are defined as surgeries requiring general, epidural or spinal anesthesia and hospitalization for more than one day.

We used propensity-score matched-pairs analyses to determine the adjusted association of preoperative renal dialysis with the primary outcome (30-day mortality). We developed a non-parsimonious multivariable logistic regression model to estimate a propensity score for preoperative renal dialysis, irrespective of outcome. Clinical significance guided the initial choice of covariates in this model: age, sex, coexisting medical conditions (included hypertension, stroke, chronic obstructive pulmonary diseases, myocardial infarction, diabetes, congestive heart failure, peripheral vascular disease, and emergency operation), teaching hospital, types of surgery, and types of anesthesia. According to a statistical research on the development of propensity score [26], we used a structured iterative approach to refine this model, with the goal of achieving covariate balance within the matched-pairs. The chi-square tests were used to measure covariate balance and a p-value <0.05 was suggested to represent meaningful covariate imbalance. We matched renal-dialysis patients to non-dialysis patients using a greedy-matching algorithm with a calliper width of 0.2 SD of the log odds of the estimated propensity score. This method could remove 98% of the bias from measured covariates [27], [28].

### Outcome Measures

We evaluated coexisting medical illnesses of study subjects including acute myocardial infarction, acute renal failure, chronic obstructive pulmonary disease, congestive heart failure, hypertension, diabetes mellitus, peripheral vascular disease and stroke within the preoperative 24-month period using medical claims data. Parameters of medical services used, such as surgeries performed in teaching hospitals or not and emergency operations, were also considered as surgical functional status. Six major postoperative complications (acute myocardial infarction, deep wound infection, pneumonia, postoperative bleeding, septicemia and stroke) and subsequent overall in-hospital mortality within 30 days after index surgery were the study’s primary outcomes [29]. Information collected on surgical admission included length of hospital and intensive care unit stays. We considered the patients in specific groups who’s length of hospitalization were above the lower limit of the highest quintile of length of stay (≥18 days in this study) among the overall patients and divided by number of patients in each group as the percent of increased length of stay. The same definition was applied to percentage of increased medical expenditures. The ICU stay and increased length of stay were also identified as adverse outcomes. According to the *International Classification of Diseases, 9th Revision, Clinical Modification* (ICD-9-CM) we defined comorbidities and postoperative complications including hypertension (ICD-9-CM 401–405), chronic obstructive pulmonary disease (ICD-9-CM 490–496), diabetes mellitus (ICD-9-CM 250), stroke (ICD-9-CM 430–438), acute myocardial infarction (ICD-9-CM 410), congestive heart failure (ICD-9-CM 428), peripheral vascular disease (ICD-9-CM 443), postoperative bleeding (ICD-9-CM 998.0, 998.1 and 998.2), pneumonia (ICD-9-CM 480–486), septicemia (ICD-9-CM 038, 998.5) and deep wound infection (ICD-9-CM 958).

To assess dialysis type-specific effects on postoperative complications and mortality, we categorized preoperative dialysis into HD and PD. The differential effects of preoperative hypertension and diabetes mellitus and of numbers of coexisting medical conditions on postoperative complications and mortality were also evaluated among surgical patients receiving dialysis.

### Data Analysis

We compared postoperative complication and mortality rates between surgical patients with and without preoperative dialysis using Chi-square tests and descriptive parameters concerning demographic status, coexisting medical conditions, operation in teaching hospital or not, and types of surgery and anesthesia. Odds ratios (ORs) and 95% confidence intervals (CIs) for increased length of stay, ICU stay, 30-day postoperative complications and mortality associated with preoperative dialysis (HD or PD) were estimated in multivariate logistic regressions by adjusting operation in teaching hospital or not, preoperative coexisting medical conditions, and types of surgery and anesthesia. We also calculated the adjusted ORs and 95% CIs for surgical adverse outcomes associated with number of coexisting diseases and the effects of hypertension and diabetes. All analyses were performed using SAS statistical software (version 9.1 for Windows; SAS Institute). The results were considered statistically significant when 2-tailed p<0.05.

## Results

Under propensity-score matched method, there was no significant difference in sociodemographic factors, co-existing medical conditions, types of surgery and types of anesthesia between patients with and without dialysis (Table 1). Compared with controls, patients with preoperative regular dialysis showed higher rates of postoperative acute myocardial infarction (1.23% vs. 0.69%, p<0.0001), pneumonia (7.23% vs. 4.73%, p<0.0001), bleeding (4.92% vs. 3.55%, p<0.0001), septicemia (10.98% vs. 4.46%, p<0.0001), overall postoperative complication rate (25.95% vs. 18.44%, p<0.0001) and significantly higher 30-day postoperative mortality rate (2.64% vs. 0.85%, p<0.0001) (Table 2). Incidence of increased length of hospitalization, ICU stay, and increased medical expenditure was higher in dialysis patients than in non-dialysis patients. After adjustment for age, sex, teaching hospital, coexisting disease and type of surgery and anesthesia, surgical patients with preoperative regular dialysis exhibited significantly higher risk of postoperative complications including acute myocardial infarction (OR 1.85; 95% CI 1.35 to 2.54), pneumonia (OR 1.70, 95% CI 1.49 to 1.94), postoperative bleeding (OR 1.40, 95% CI 1.21 to 1.63), septicemia (OR 2.83, 95% CI 2.50 to 3.20) and overall complications (OR 1.70, 95% CI 1.58 to 1.84). The corresponding OR for 30-day postoperative mortality was 3.33 (95% CI 2.56 to 4.33). The ORs of preoperative dialysis associated with increased length of stay, ICU stay and increased medical expenditure were 2.20 (95% CI 2.03 to 2.38), 2.37 (95% CI 2.20 to 2.55), and 2.91 (95% CI 2.68 to 3.16), respectively.

**Table 1:** Characteristics of surgical patients with renal dialysis and controls.*

	Controls,N = 8937		Dialysis,N = 8937			p
Sex	n	(%)	n	(%)		1.00
Female	4892	(54.7)	4892	(54.7)		
Male	4045	(45.3)	4045	(45.3)		
Age, years						0.34
20–29	136	(1.5)	161	(1.8)		
30–39	425	(4.8)	450	(5.0)		
40–49	1157	(13.0)	1193	(13.4)		
50–59	2044	(22.9)	2080	(23.3)		
60–69	2296	(25.7)	2286	(25.6)		
70–79	2148	(24.0)	2093	(23.4)		
≥80	731	(8.2)	674	(7.5)		
Operation in teaching hospital						0.34
No	329	(3.7)	353	(4.0)		
Yes	8608	(96.3)	8584	(96.0)		
Coexisting medical conditions						
Hypertension	7558	(84.6)	7518	(84.1)	0.41	
Stroke	1654	(18.5)	1637	(18.3)	0.74	
COPD	3276	(36.7)	3268	(36.6)	0.90	
Myocardial infarction	3965	(44.4)	3930	(44.0)	0.59	
Diabetes	4628	(51.8)	4589	(51.4)	0.55	
Congestive heartfailure	3387	(37.9)	3399	(38.0)	0.85	
Peripheral vascular disease	692	(7.7)	730	(8.2)	0.29	
Emergency operation	455	(5.1)	507	(5.7)	0.08	
Types of surgery					0.91	
Skin	580	(6.5)	604	(6.8)		
Breast	75	(0.8)	82	(0.9)		
Musculoskeletal	2768	(31.0)	2750	(30.8)		
Respiratory	388	(4.3)	382	(4.3)		
Digestive	1871	(20.9)	1888	(21.1)		
Kidney, ureter, bladder	1269	(14.2)	1283	(14.4)		
Delivery, CS, abortion	28	(0.3)	28	(0.3)		
Neurosurgery	764	(8.6)	709	(7.9)		
Eye	155	(1.7)	171	(1.9)		
Others	1039	(11.6)	1040	(11.6)		
Types of anesthesia					0.43	
General	6872	(76.9)	6828	(76.4)		
Epidural or spinal	2065	(23.1)	2109	(23.6)		

* Renal dialysis: at least three times per week lasting more than three months for dialysis therapy within the 24-month preoperative period.

COPD, chronic obstructive pulmonary disease; CS, caesarean section.

**Table 2:** Risk of 30-day postoperative mortality and complications among controls and patients with preoperative regular dialysis receiving non-cardiac surgery.

			Multivariate*	
Adverse outcomes	Control, %	Dialysis, %	OR	(95% CI)
30-day postoperative mortality	0.85	2.64	3.33	(2.56–4.33)
Postoperative complications				
Acute myocardial infarction	0.69	1.23	1.85	(1.35–2.54)
Deep wound infection	0.85	0.54	0.62	(0.43–0.89)
Pneumonia	4.73	7.23	1.70	(1.49–1.94)
Postoperative bleeding	3.55	4.92	1.40	(1.21–1.63)
Septicemia	4.46	10.98	2.83	(2.50–3.20)
Stroke	8.11	7.43	0.94	(0.83–1.06)
Any of above	18.44	25.95	1.70	(1.58–1.84)
Increased length of stay†	13.92	24.57	2.20	(2.03–2.38)
ICU stay	20.06	33.47	2.37	(2.20–2.55)
Elevated medical expenditure	12.58	27.41	2.91	(2.68–3.16)

* Adjusted for age, sex, teaching hospital, coexisting disease and type of surgery and anesthesia.

† Increased length of stay: patients in specific groups who’s length of hospitalization were above the lower limit of the highest quintile of length of stay (≥18 days in this study) among the overall patients and divided by number of patients in each group as the percent of increased length of stay.

OR, odds ratio; CI, confidence interval; ICU, intensive care unit.

Considering 30-day postoperative mortality among patients receiving different types of renal dialysis, surgical patients had higher risk when receiving regular preoperative PD (OR 2.71, 95% CI 1.70 to 4.31) or HD (OR 3.42, 95% CI 2.62 to 4.47) when compared with surgical patients without dialysis. As for postoperative complications, preoperative HD or PD significantly increased risk of postoperative pneumonia, postoperative bleeding, septicemia and overall complications without significant differences between PD and HD (Table 3). HD patients had no significant higher mortality after surgery compared with PD patients (OR = 1.26, 95% CI 0.82–1.94) (not showed in the tables).

**Table 3:** Risk of postoperative 30-day mortality and complications among surgical patients with peritoneal or hemodialysis versus controls.*

	Controls		Peritoneal dialysis		Hemodialysis	
Adverse outcomes	OR	(95% CI)	OR	(95% CI)	OR	(95% CI)
30-day postoperative mortality	1.00	(reference)	2.71	(1.70–4.31)	3.42	(2.62–4.47)
Postoperative complications						
Acute myocardial infarction	1.00	(reference)	1.63	(0.87–3.06)	1.88	(1.37–2.59)
Deep wound infection	1.00	(reference)	0.28	(0.09–0.90)	0.67	(0.46–0.97)
Pneumonia	1.00	(reference)	1.73	(1.34–2.23)	1.70	(1.48–1.94)
Postoperative bleeding	1.00	(reference)	2.29	(1.82–2.87)	1.24	(1.06–1.45)
Septicemia	1.00	(reference)	2.73	(2.20–3.40)	2.85	(2.51–3.23)
Stroke	1.00	(reference)	0.96	(0.74–1.24)	0.93	(0.82–1.06)
Any of above	1.00	(reference)	1.86	(1.61–2.15)	1.68	(1.55–1.82)
Increased length of stay	1.00	(reference)	2.31	(1.99–2.68)	2.18	(2.01–2.37)
ICU stay	1.00	(reference)	2.27	(1.98–2.61)	2.38	(2.20–2.57)
Elevated medical expenditure	1.00	(reference)	2.78	(2.41–3.22)	2.94	(2.70–3.19)

* Adjusted for age, sex, teaching hospital, coexisting disease and type of surgery and anesthesia.

† The highest quintile of length of stay during the surgical admission (the patient percentage for each group belonging to the highest quintile of length of stay [≥18 days] of the total patients as percentage of increased length of stay).

OR, odds ratio; CI, confidence interval; ICU, intensive care unit.

Among patients with regular dialysis, those who had both hypertension and diabetes were at the highest risk of 30-day postoperative pneumonia (OR 1.68, 95% CI 1.19 to 2.38), septicemia (OR 1.45, 95% CI 1.09 to 1.93), stroke (OR 1.89, 95% CI 1.27 to 2.81) and overall major complications (OR 1.74, 95% CI 1.43 to 2.13) compared with patients without diabetes or hypertension (Table 4). The highest risks of increased length of stay (OR 1.78, 95% CI 1.46 to 2.17), ICU stay (OR 1.22, 95% CI 1.02 to 1.44) and increased medical expenditure (OR 1.44, 95% CI 1.21 to 1.73) were also found in patients with hypertension and diabetes compared with patients without diabetes or hypertension.

**Table 4:** Postoperative mortality and complications associated with hypertension or diabetes mellitus in surgical patients with preoperative regular dialysis.*

	Hypertension		No		Yes		No		Yes
	Diabetes		No		No		Yes		Yes
	OR	(95% CI)	OR	(95% CI)	OR	(95% CI)	OR	(95% CI)	
Postoperative 30-day mortality	1.00	(reference)	0.93	(0.56–1.55)	1.48	(0.68–3.25)	1.09	(0.65–1.81)	
Postoperative 30-day complications									
Acute myocardial infarction	1.00	(reference)	1.11	(0.45–2.75)	1.40	(0.34–5.70)	1.82	(0.76–4.39)	
Deep wound infection	1.00	(reference)	1.57	(0.35–7.15)	2.57	(0.35–18.9)	2.53	(0.57–11.3)	
Pneumonia	1.00	(reference)	1.17	(0.83–1.66)	1.48	(0.84–2.59)	1.68	(1.19–2.38)	
Postoperative bleeding	1.00	(reference)	1.51	(1.03–2.20)	1.45	(0.76–2.76)	1.50	(1.01–2.22)	
Septicemia	1.00	(reference)	1.06	(0.79–1.41)	1.53	(0.98–2.39)	1.45	(1.09–1.93)	
Stroke	1.00	(reference)	1.89	(1.27–2.79)	1.54	(0.80–2.95)	1.89	(1.27–2.81)	
Any of above	1.00	(reference)	1.32	(1.08–1.61)	1.58	(1.14–2.20)	1.74	(1.43–2.13)	
Increased length of stay†	1.00	(reference)	1.04	(0.85–1.27)	1.62	(1.18–2.24)	1.78	(1.46–2.17)	
ICU stay	1.00	(reference)	0.94	(0.80–1.11)	1.24	(0.92–1.67)	1.22	(1.02–1.44)	
Elevated medical expenditure	1.00	(reference)	1.03	(0.87–1.23)	1.47	(1.08–2.01)	1.44	(1.21–1.73)	

* Adjusted for age, sex, teaching hospital, coexisting disease and type of surgery and anesthesia.

† The highest quintile of length of stay during the surgical admission (the patient percentage for each group belonged to the highest quintile of length of stay [≥18 days] of the total patients as percentage of increased length of stay).

OR, odds ratio; CI, confidence interval; ICU, intensive care unit.

Among surgical patients with preoperative renal dialysis, the number of preoperative co-existing medical illnesses was associated with postoperative adverse outcomes including postoperative bleeding, septicemia, stroke and overall complications. The risks of increased length of hospitalization, ICU stay and increased medical expenditure were positively correlated with the number of preoperative co-existing diseases among surgical patients with preoperative renal dialysis (Table 5).

**Table 5:** Postoperative mortality and complications associated with additional coexisting diseases in surgical patients with preoperative regular dialysis.

	Number of co-existing diseases*									
	0		1		2		3		4	
	OR	(95% CI)	OR	(95% CI)	OR	(95% CI)	OR	(95% CI)	OR	(95% CI)
Postoperative 30-day mortality	1.00	(reference)	0.82	(0.38–1.76)	1.24	(0.62–2.48)	0.94	(0.46–1.91)	1.47	(0.75–2.89)
Postoperative 30-day complications										
Acute myocardial infarction	1.00	(reference)		NA‡		NA‡		NA‡		NA‡
Deep wound infection	1.00	(reference)		NA‡		NA‡		NA‡		NA‡
Pneumonia	1.00	(reference)	0.73	(0.46–1.15)	0.70	(0.45–1.08)	1.04	(0.69–1.58)	1.35	(0.91–2.02)
Postoperative bleeding	1.00	(reference)	1.66	(0.96–2.86)	2.04	(1.20–3.46)	1.61	(0.94–2.77)	2.12	(1.25–3.58)
Septicemia	1.00	(reference)	1.40	(0.89–2.20)	1.56	(1.01–2.40)	1.90	(1.24–2.91)	2.63	(1.73–3.99)
Stroke	1.00	(reference)	1.26	(0.74–2.15)	1.45	(0.87–2.40)	2.27	(1.38–3.74)	2.83	(1.74–4.60)
Any of above	1.00	(reference)	1.30	(0.98–1.72)	1.42	(1.08–1.86)	1.87	(1.43–2.44)	2.61	(2.01–3.39)
Increased length of stay†	1.00	(reference)	1.12	(0.84–1.48)	1.26	(0.96–1.65)	1.66	(1.27–2.17)	2.59	(2.00–3.36)
ICU stay	1.00	(reference)	0.90	(0.72–1.13)	0.99	(0.80–1.23)	1.21	(0.97–1.50)	1.54	(1.25–1.91)
Elevated medical expenditure	1.00	(reference)	1.12	(0.89–1.41)	1.08	(0.86–1.36)	1.32	(1.05–1.65)	1.83	(1.47–2.28)

* Adjusted for age, sex, teaching hospital, coexisting disease and types of surgery and anesthesia.

† The highest quintile of length of stay during the surgical admission (patient percentage for each group belonging to the highest quintile of length of stay [≥18 days] of the total patients as percentage of increased length of stay).

‡ NA, Not available due to small sample size.

OR, odds ratio; CI, confidence interval; ICU, intensive care unit; NA, not available.

## Discussion

After adjusting the covariates under propensity-score matched method in this large-scale population-based study, ESRD patients with preoperative dialysis therapy, either PD or HD, encountered significantly higher postoperative complication and mortality rates compared with controls when receiving non-cardiac surgery, and facing significantly high risks of acute myocardial infarction, pneumonia, bleeding and septicemia. Compared with dialysis patients with neither hypertension nor diabetes mellitus, dialysis patients with hypertension and diabetes mellitus were found to have the highest relative risks for postoperative complications among groups. Increasing numbers of medical conditions were linked with incremental increases in rates for overall postoperative complications, and medical utility when compared with dialysis patients without additional comorbidities.

Discrepancies were noted between the risks for systemic infection (pneumonia and septicemia) and deep wound infection among patients under dialysis management in our data and previous studies [19], [20]. This might be explained by recent improvements in restrictive fluid management for surgical patients under dialysis therapy. During the last decade, worldwide acceptance of restrictive fluid regimens applied to surgical patients receiving major operations such as thoracic surgery resulted in significant decreases in pulmonary morbidity [30], [31]. The restrictive fluid strategy for surgical patients further provided better outcomes than liberal fluid regimen in overall postoperative morbidity [32]. Cardiopulmonary and tissue-healing complications were also significantly reduced under a limited fluid administration regimen for patients receiving elective colorectal surgeries [33]. Most patients would receive dialysis treatment followed by restriction of perioperative fluid before elective surgery, and surgeons keep patients in a relatively dehydrated status to limit the potential risk of local/deep wound infection [34]. However, further prospective study is needed for an evidence-based explanation.

When kidney dysfunction progresses to ESRD, patients must choose a type of renal dialysis, PD or HD, for replacement therapy depending on patients’ preference, economic status, geographic location and severity of comorbid illnesses [35]. Although the patients maintained on HD seem to have a higher comorbid burden than those on PD, outcome benefits were still equivocal [35]–[38]. The impact of different types of renal dialysis on surgical adverse outcomes in large scale has not been documented previously. After adjustment of patients’ demographics and comorbidities with propensity-score matched-pair controls, we demonstrated that surgical patients under HD had relatively higher risk of in-hospital mortality than the PD group when compared with non-dialysis controls. As for postoperative complications, patients with HD showed similar risks in overall complications when compared with patients with PD except for acute myocardial infarction.

Comorbidities may predispose uremic patients to higher mortality and morbidity [39]. However, the impact of complex pre-existing comorbidities, as shown by numbers or types, on surgical outcomes in ESRD patients had not been well demonstrated. In our data, surgical dialysis patients with hypertension and diabetes mellitus exhibited the highest complication rates than patients with either hypertension or diabetes or neither. According to the 2005 annual data report by Taiwan’s renal registry, causes other than chronic glomerulonephritis, chronic interstitial nephritis, hypertension or diabetes mellitus constituted a high proportion, 24.6% of ESRD, in prevalent dialysis patients [10]. The disparity in etiology other than common medical conditions might partially explain the relatively lower mortality of ESRD patients in Taiwan (118.2 per thousand dialysis patients) in comparison with that in the United States (236 per thousand dialysis patients) [1], [10]. Similar conditions also exist in the Western world with environmental pollutants and drug abuse [2]–[4]. A possible explanation for this phenomenon might be attributed to etiologies other than hypertension and diabetes for this specific population. First, Orientals such as Taiwanese people were frequent users of traditional alternative medicine, and habitually received herbal remedies [3], [40]. Another reason might be chronic use of self-prescribed over-the-counter analgesics such as aspirin, acetaminophen or conventional non-steroidal anti-inflammatory drugs which were universal [2]. Long-term exposure to these medications might result in chronic kidney disease and subsequent ESRD [2], [3]. Renal impairment due to heavy metal intoxication should also be taken into consideration because environmental pollutants are ingested with water, food or herbal drugs; this is a critical health issue in Taiwan and over the world [4]. Lin et al. showed that low-level environmental lead exposure can accelerate progression of renal dysfunction in patients without diabetes mellitus or hypertension [4].

Patients with both hypertension and diabetes mellitus had higher risks over the non-hypertensive, non-diabetic group in postoperative pneumonia and septicemia, but these risks were not significantly different in patients with hypertension or diabetes alone. Patients with both hypertension and diabetes mellitus also had the significant risk in postoperative adverse outcomes. These results indicate the combination of hypertension and diabetes might have an additive influence surpassing each disease’s individual effect on postoperative outcomes, especially systemic infection (pneumonia and septicemia) and cerebrovascular events. In contrast, dialysis patients without hypertension or diabetes exhibited lower morbidity rate, and it can be explained by difference in etiological severity between diabetes/hypertension, herbal drugs, analgesics, heavy metal intoxication and etc. In our data, the combined effect of hypertension and diabetes increased the risk of deep wound infection without statistical significance and the independent association between diabetes and deep wound infection in dialysis surgical patients is still controversial.

Several limitations of this study must be addressed. First, the study is retrospective, and the database did not disclose detailed information regarding characteristics or severity of dialysis, such as specific etiology and definite duration of dialysis. The administrative database also lacked detailed profiles of dialysis management including dialysis time, type of dialysate, use of specific drugs and body weight changes. All of these factors might relate to surgical risks in patients under regular maintenance dialysis. In addition, detailed variables concerning the perioperative risks of surgery and anesthesia are not available in this database, such as preoperative laboratory data, blood pressure, oxygenation status, total blood loss, transfusions, and the use of prophylaxis antibiotics or inotropes. With such a large sample size in this study, we assumed that the influence of all of these covariates was evenly distributed between groups and bias would be diminished. Third, the study’s design and grouping of hemodialysis versus peritoneal dialysis, hypertension versus diabetes mellitus, and categorization by the numbers of comorbidities, are all procedure- or diagnosis-oriented. Thus the study can validate only the association of factors and outcomes, not causation. Finally, our retrospective study did not achieve randomized distribution between groups. In spite of meticulous adjustment of major covariates, this non-randomization might still influence risk estimations and need further investigation.

In this nationwide population-based study using Taiwan’s National Health Insurance Research Database with propensity-score matched method, we found significant increases in postoperative mortality and complication rates among surgical patients with preoperative regular dialysis underwent non-cardiac surgeries, either HD or PD. Increasing numbers of comorbidities, including hypertension or diabetes, may predispose these patients to higher rates of postoperative complications. Our findings suggested that meticulous preoperative assessment, optimal control for diabetes and hypertension, early recognition of morbidities and appropriate interventions might reduce adverse outcomes in dialysis patients receiving non-cardiac surgery.
